# Ascites induces modulation of *α*6*β*1 integrin and urokinase plasminogen activator receptor expression and associated functions in ovarian carcinoma

**DOI:** 10.1038/sj.bjc.6602495

**Published:** 2005-03-29

**Authors:** N Ahmed, C Riley, K Oliva, G Rice, M Quinn

**Affiliations:** 1 Gynaecological Cancer Research Centre, Royal Women's Hospital, Melbourne, Australia; 2 Department of Obstetrics and Gynaecology, University of Melbourne, Melbourne, Australia; 3 Translational Proteomics Division, Baker Heart Research Institute, Melbourne, Australia

**Keywords:** integrin, urokinase plasminogen activator receptor, ovarian cancer, plasminogen activation, ascites and metastasis

## Abstract

Interactions between cancer cells and the surrounding medium are not fully understood. In this study, we demonstrate that ascites induces selective changes in the expression of integrins and urokinase plasminogen activator/urokinase plasminogen activator receptor (uPA/uPAR) in ovarian cancer cells. We hypothesise that this change of integrin and uPA/uPAR expression triggers signalling pathways responsible for modulating phenotype-dependent functional changes in ovarian cancer cells. Human ovarian surface epithelial (HOSE) cell lines and epithelial ovarian cancer cell lines were treated with ascites for 48 h. Ascites induced upregulation of *α*6 integrin, without any change in the expression of *α*v, *β*1 and *β*4 integrin subunits. Out of the four ovarian cancer cell lines studied, ascites induced enhancement in the expression of uPA/uPAR in the more invasive OVCA 433 and HEY cell lines without any change in the noninvasive OVHS1 and moderately invasive PEO.36 cell lines. On the other hand, no change in the expression of *α*6 integrin or uPAR, in response to ascites, was observed in HOSE cells. In response to ascites, enhancement in proliferation and in adhesion was observed in all four ovarian cancer cell lines studied. In contrast, no significant increase in proliferation or adhesion by ascites was observed in HOSE cells. Ascites-induced expression of uPA/uPAR correlated with the increased invasiveness of HEY and OVCA 433 cell lines but was not seen in OVHS1, PEO.36 and HOSE cell lines. Upregulation of *α*6 integrin and uPA/uPAR correlated with the activation of Ras and downstream Erk pathways. Ascites-induced activation of Ras and downstream Erk can be inhibited by using inhibitory antibodies against *α*6 and *β*1 integrin and uPAR, consistent with the inhibition of proliferation, adhesion and invasive functions of ovarian cancer cell lines. Based on these findings, we conclude that ascites can induce selective upregulation of integrin and uPA/uPAR in ovarian cancer cells and these changes may modulate the functions of ovarian carcinomas.

Cancer is a clonal phenomenon initiated by mutation in normal cells that results in the generation of clones with diversified phenotype (Greaves and Greaves, 2002). The growth of tumour results in the dominance of proliferation of cancer cells compared to normal cells. As the tumour grows, some cancer cells can become genetically unstable and they may progressively acquire aggressive properties that subsequently enable them to invade the adjacent tissues, followed by systemic spreading, leading to fatal outcome when left unchallenged (Greaves and Greaves, 2002). Tumour microenvironment may have a differential effect on the diversified clonal population of cancer cells enabling them to respond to the environment in a phenotype-dependent manner (Fidler, 2002).

Ascites is a common and distressing complication of ovarian cancer (Hirabayashi and Graham, 1970). Ascites contains growth factors and other extracellular matrix constituents produced by tumour-infiltrating leucocytes and noncancerous activated mesothelial cells (Offner *et al*, 1995). The shedding of malignant cells from the surface of the ovarian tumour into the peritoneal cavity is a common scenario in the progression of ovarian carcinoma. Subsequent implantation of these shed tumour cells to secondary sites along the mesothelial lining of the peritoneum depends on their proliferation, adhesion, migration and invasive phenotype. Ovarian cancer cells have the ability to survive in the peritoneal tumour fluid or ascites as a single cell or small clumps of aggregated cells or as spheroids. Hence, a greater understanding of the biological changes induced by ascites to the cancer cells is vital to understand the biology of ovarian cancer progression.

Integrins are a family of heterodimeric trans-membrane receptors that regulate cell–cell and cell–matrix interaction (Hynes, 1992). In ovarian carcinoma, integrins have been shown to mediate the organisation of extracellular matrix (ECM) (Carreiras *et al*, 1999a), adhesion to ECM components (Lessan *et al*, 1999) and cell motility (Carreiras *et al*, 1999b). The *β*1 family of integrins and CD44 has been shown to mediate interactions between ovarian carcinoma cells and the mesothelial cells that line the abdominal organs (Lessan *et al*, 1999). CD44 binds the ECM glycosaminoglycan hyaluronan with high affinity (Casey and Skubitz, 2000) and affects cell adhesion (Lessan *et al*, 1999), migration (Casey and Skubitz, 2000) and tumour growth (Strobel *et al*, 1997) in ovarian carcinoma cells.

One critical pathway frequently implicated in cancer growth, invasion and spread is the plasminogen (Plg) activation cascade (Blasi, 1999). Activation of this cascade results in the formation of cell-surface plasmin (Pn) that is essential for the degradation of the ECM in surrounding tissue barrier (Blasi, 1999). In ovarian cancer, a strong correlation has been reported between urokinase plasminogen activator (uPA) antigen levels and poor prognosis (Konecny *et al*, 2001) and uPA is localised in primary and metastatic ovarian cancer specimens, as well as in the conditioned medium of many (but not all) ovarian cancer cell lines (Ahmed *et al*, 2002b). In addition, ovarian cancer patient's tumour fluid expresses a higher concentration of cell-free urokinase plasminogen activator receptor (uPAR) (Pedersen *et al*, 1993) and high levels of cell-associated uPA correlate with advance stage disease (Tecimer *et al*, 2000).

One of the consequences of integrin ligation is activation of Ras and its downstream Erk MAP kinase pathway that participates in proliferative responses to multiple stimuli (Clark and Brugge, 1995). In addition, elevated uPAR expression in cancer cells is maintained by constitutively activated Erk MAP kinase activity (Lengyel *et al*, 1997), and recently it has been shown that blockade of the Erk MAP kinase pathway suppresses growth of colonic and ovarian tumours *in vivo* (Sebolt-Leopold *et al*, 1999). Collectively, these data indicate that integrins in association with uPAR can sustain activation of the Ras pathway to regulate proliferation and proteolytic function of cancer cells.

The purpose of this study was to elucidate the biological role of ascites in regulating integrin-mediated changes in ovarian cancer growth and function. We examined selective changes in the expression of integrins and uPA/uPAR in HOSE and ovarian cancer cell lines in response to ascites. We show that *α*6*β*1 integrin and uPA/uPAR participate in ascites-mediated functional changes of ovarian cancer cells. Our findings suggest that ascites can induce markedly different functional outcomes in ovarian carcinomas and that these differences can help one to understand biological processes *in vivo* for better translation of responses to treatment.

## MATERIALS AND METHODS

### Cell lines

The epithelial ovarian cancer cell lines HEY and PEO.36 were obtained from Dr Georgia Chenevix-Trench, Queensland Institute for Medical Research, Australia; the clear cell carcinoma cell line OVHS 1 was from Dr Hideki Sakamoto, Nihon University School of Medicine, Japan and epithelial ovarian cancer cell line OVCA 433 was from Dr Robert Bast, MD Anderson Centre, Houston, USA. HOSE cells immortalised by transfection with SV-40 antigen (Auersperg *et al*, 1999) were obtained from Professor Nelly Auersperg, University of British Columbia, Vancouver, Canada. HOSE cells were maintained in Medium 199/MCDB (1 : 1), while ovarian cancer cell lines were maintained in RPMI 1640. Each medium was supplemented with 10% heat-inactivated fetal bovine serum (FBS), 10 mM HEPES, 100 *μ*g ml^−1^ of penicillin and streptomycin. Cells were incubated at 37°C in 5% CO_2_ and routinely checked for contamination. Viability was checked routinely by Trypan blue exclusion method.

### Antibodies and reagents

Monoclonal antibodies against *α*6 (ASC-6), *α*v (AV1), *β*1(P5D2) and *β*4(439-9B) integrin were obtained from Chemicon International (CA, USA). Monoclonal antibodies against uPA (394) and uPAR (3936) were from American Diagnostica (Greenwich, USA), anti-phospho-Erk from New England Biolab (MA, USA) and tubulin was from Santa Cruz (CA, USA). Phycoerythrin-conjugated goat anti-mouse IgG was obtained from Chemicon International (CA, USA) and peroxidase-conjugated goat anti-mouse antibody was from Bio-Rad (CA, USA).

The study was approved by the Research and Human Ethics Committee (HEC#02/30 02/29) of Royal Women's Hospital, Melbourne, Australia. Ascites was obtained from patients diagnosed with Stage 2 or Stage 3 ovarian cancer, admitted to the Oncology Dysplasia Unit of the Royal Women's Hospital, Melbourne. Two staff pathologists in the hospital performed the pathology diagnosis of these patients. These patients were diagnosed with serous cystadenocarcinoma and endometrioid carcinoma. Protein content of ascites varied between 30 and 40 mg ml^−1^.

### Immunohistochemistry

Resected tissues not required for clinical analyses were obtained from patients who presented for surgery at the Royal Women's Hospital, Melbourne, after the provision of a participant information statement and only with informed consent. Normal ovaries, needed for control comparisons, were removed from patients undergoing surgery as a result of suspicious ultrasound images, palpable abdominal masses and family history. Histological grading of ovarian carcinoma was performed by the method described by Silverberg (2000).

The tissue was frozen in embedding medium (OCT) by immersion in isopentane cooled in dry ice. It was then stored at −80°C until needed. Frozen sections of the tissue were cut at 5 *μ*m thickness and if not used immediately, stored at −20°C. For staining, sections were fixed in cold acetone for 15 min and held in Tris buffer (100 mM, pH 7.6). Endogenous peroxidase activity was removed using 3% hydrogen peroxide in methanol, and endogenous biotin activity was blocked using diluted egg white (5% in distilled water) and skim milk powder (5% in distilled water) for 10 min each. The sections were incubated for 1 h in primary antibody diluted 1 : 200 in 1% BSA in Tris buffer (100 mM, pH 7.6). Antibody binding was amplified using biotin and streptavidin HRP (Chemicon, CA, USA) for 15 min each and the complex visualised using diaminobenzidine (DAB). Nuclei were lightly stained with Mayer's haematoxylin. An isotype IgG1, suitably diluted, was substituted for the antibody as a negative control.

Sections were assessed microscopically for positive DAB staining. The extent of staining of integrin and uPAR expression was scored in a blind fashion as 0 (<10%), 1 (11–25%), 2 (26–50%), 3 (51–75%), 4 (76–90%) and 5 (>90%) cells. Four sections were assessed per tissue. In addition to the extent of staining, tissue and cellular distribution of staining was determined. Parallel frozen sections were stained with hematoxylin and eosin to confirm the pathology diagnosis. Hematoxylin and eosin and immunostained sections were reviewed independently by two pathologists to verify cell type, grade and immunohistochemical score.

### Flow cytometric analyses

Monolayer cultures of HOSE and ovarian cancer cell lines in serum-free medium were treated with ascites (3 mg ml^−1^) or 10% fetal calf serum (3 mg ml^−1^) for 48 h. Cells were washed twice with PBS, harvested with trypsin-versene (CSL Biosciences, Australia) and 10^6^ cells incubated with primary antibody for 30 min at 4°C and washed twice with PBS. Cells were stained with secondary antibody conjugated with phycoerythrin for 20 min at 4°C, washed twice with phosphate-buffered saline (PBS) and then resuspended in 0.5 ml PBS prior to FACScan analysis (Becton Dickinson, NJ, USA). All data were analysed using Cell Quest software (Becton-Dickinson). Results are expressed as mean fluorescence units for triplicate determinations.

### Ras activation assay

Ras activation assay was performed as described in the manufacturer's instruction of the kit provided by Upstate Biotechnology (NY, USA). Briefly, exponentially growing ovarian cancer cell lines were serum starved for 16 h, and then incubated in serum-free medium±ascites (3 mg ml^−1^) for 30 min. Cells were harvested, lysed in lysis buffer (125 mM HEPES, pH 7.5, 150 mM NaCl, 1% NP 40, 50 mM MgCl_2_, 5 mM EDTA and 10% glycerol) and supernatant prepared by centrifugation for 20 min at 4°C at 900 **g**. Ras activation assay was performed on cell lysates containing equal amounts of protein. The assay method involves immunoprecipitation of Ras-GTP and Ras-GDP from the cell lysates treated with GTP*γ*S and GDP*γ*S. The immunoprecipitates were detected by Western immunoblot using Ras monoclonal antibody.

### Immunoprecipitation and coimmunoprecipitation analyses

Monolayer cultures of ovarian cancer cell lines were washed twice with PBS and harvested with trypsin-versene. To detect cell surface level of integrins, cells were lysed in lysis buffer (100 mM Tris-HCl, pH 7.5, 150 mM NaCl, 1 mM CaCl_2_, 1% Triton X-100, 0.1% SDS, 0.1% NP-40, 1 mM vanadate, 1 *μ*g ml^−1^ pepstatin, 1 mM PMSF, 5 *μ*g ml^−1^ Trasylol and 1 *μ*g ml^−1^ of leupeptin). Protein concentration of the cell lysates was determined and lysates containing equal protein were used for immunoprecipitation. Cells lysates were immunoprecipitated with mAbs against *α*6 (ASC-6), *β*1 (PD52), *β*4 (439-9B) integrins or isotype-matched control. Samples were resolved in 7.5% SDS–PAGE gel under nonreducing conditions and were transferred to nitrocellulose membranes. Membranes were probed with the respective integrin antibodies followed by peroxidase-labelled secondary antibody.

### Western blotting

Exponentially growing HOSE and ovarian cancer cell lines were serum starved for 16 h. Cells were incubated in serum-free medium±ascites (3 mg ml^−1^) for 30 min. Cells were harvested, lysed in lysis buffer (125 mM HEPES, pH 7.5, 150 mM NaCl, 1% NP 40, 50 mM MgCl_2_, 5 mM EDTA and 10% glycerol) and supernatant prepared by centrifugation for 20 min at 4°C at 900 **g**. Cell lysates containing equal amounts of protein were electrophoresed on 10 or 18% SDS–PAGE gels under nonreducing or reducing conditions and transferred to nitrocellulose membranes. Membranes were probed with primary antibody followed by peroxidase-labelled secondary antibody and visualised by the ECL (Amersham, UK) detection system according to the manufacturer's instructions.

### Cell proliferation assay

In total, 1 × 10^5^ cells were plated in the presence and absence of (3 mg ml^−1^) FBS or ascites (3 mg ml^−1^) in 24-well plates. After 24 h, 0.5 *μ*Ci of [^3^H]thymidine was added to each well and cells were incubated at 37°C with 5% CO_2_ for an additional 16 h. Cells were washed with PBS, harvested and lysed in 1% Triton and measured by liquid scintillation counting.

### 3D collagen cell proliferation assay

In order to assess if the proliferation of ovarian cancer cells in response to ascites on monolayer cultures correlates with that in 3D cultures that mimics *in vivo* situation, proliferation assays were performed by 3D collagen cell culture system (Chemicon International, CA, USA). Briefly, 1 × 10^4^ cells were mixed with chilled collagen solution and plated onto a 96-well plate (Nunc, USA). The cells were incubated at 37°C for 60 min to initiate polymerisation of the collagen. After the formation of the collagen gel, 200 *μ*l of growth medium was added to each well and cells were incubated at 37°C with CO_2_ for 4 days with change of growth medium every day. [^3^H]thymidine (0.5 *μ*Ci) was added to the wells for 16 h. Cells were removed from the collagen gels by digestion using *Clostridium histolyticum* collagenase (100 *μ*g ml^−1^) for 30 min at 37°C. Cells were collected by centrifugation, washed with PBS and dissolved in 1% Triton and measured by liquid scintillation counting.

### Invasion assay

The invasive response of HOSE and ovarian cancer cells in response to ascites was determined by using Matrigel-coated invasion chambers (Becton and Dickinson, USA) as described previously (Ahmed *et al*, 2003a). Cells were grown in the presence or absence of ascites (3 mg ml^−1^) for 24 h. Approximately 2 × 10^5^ cells were incubated in Matrigel-coated inserts in serum-free medium. FBS (5%) was used as chemoattractant at 37°C for 36 h. Matrigel inserts were removed and 0.5 *μ*Ci of [^3^H]thymidine was added to the wells for 16 h. Cells were washed with PBS, dissolved in 1% Triton and measured by liquid scintillation counting.

### Adhesion assay

HOSE and ovarian cancer cell lines were treated with ascites (3 mg ml^−1^) for 24 h. In all, 1 × 10^5^ cells were plated on 96-well plates in triplicate at 37°C for 90 min. Cells were then washed three times with PBS to remove nonadhering cells and the adherent cells were fixed with 100% methanol for 5 min at room temperature. Cells were stained with 0.5% crystal violet for 15 min. Stained cells were washed with PBS, dried and absorbance was measured at 595 nm with *V*_max_ plate reader (Bio-Rad, CA, USA).

### Statistical analysis

The association between the extent of *α*6 integrin immunohiostochemical staining and histological grade of tumour was determined by *χ*^2^ analyses using the SPSS statistical package (Coakes, 1996). For uPAR immunohistochemistry, Student's *t*-test was used to test the association between the extent of staining and the histological grade of the tumour. Student's *t*-test was also used for statistical analyses of proliferation, adhesion and invasion assays. Statistical significance was indicated by *P*<0.05. Data are presented as mean±s.e.m. Each experiment was repeated 3 times and was carried out in triplicate.

## RESULTS

### *α*6, *β*1 and *β*4 integrin and uPA/uPAR profile of HOSE and ovarian cancer cells in response to ascites

**Figure 1:**
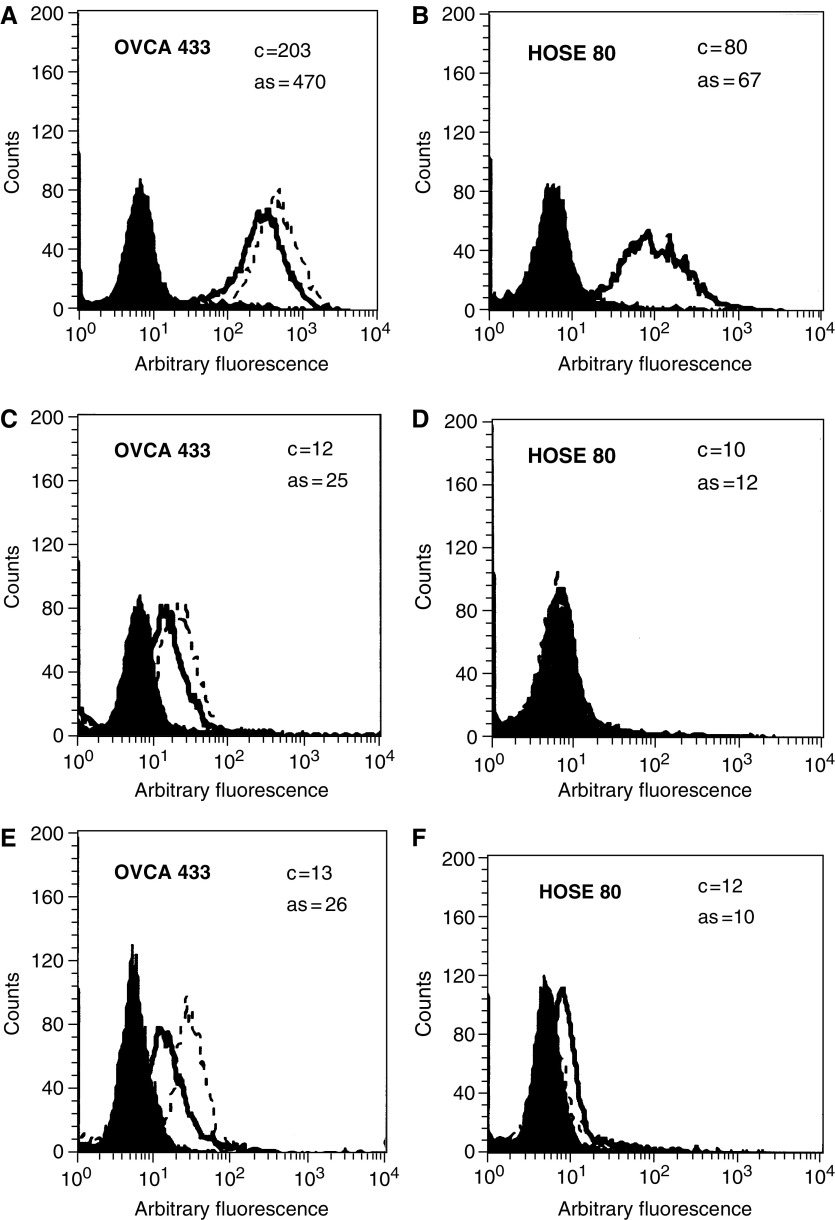
Flow cytometric analyses of (**A**, **B**) *α*6 integrin; (**C**, **D**) uPAR and (**E**, **F**) uPA in OVCA 433 and HOSE 80 cell lines in response to ascites (3 mg ml^−1^). The median intensity of fluorescence was measured (MIF, arbitrary units, log scale) and is shown in the inserts of each cell line. The filled histogram in each figure is of control IgG, black lines indicate the expression of protein in the absence of ascites (**C**), while broken lines demonstrate protein expression in the presence of ascites (as) after 48 h. Results are representative of five experiments.

**Figure 2:**
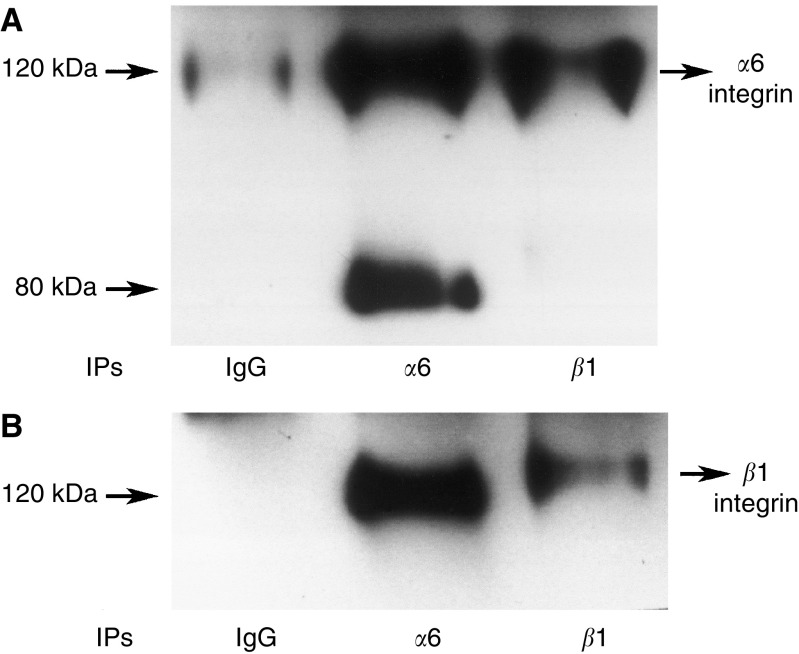
*α*6*β*1 interaction in HEY cells. Monolayer cultures of HEY cancer cell line was washed twice with PBS and harvested with trypsin-versene. Cells were lysed in lysis buffer (100 mM Tris-HCl, pH 7.5, 150 mM NaCl, 1 mM CaCl_2_, 1% Triton X-100, 0.1% SDS, 0.1% NP-40, 1 mM vanadate, 1 *μ*g ml^−1^ pepstatin, 1 mM PMSF, 5 *μ*g ml^−1^ Trasylol and I *μ*g ml^−1^ of leupeptin). Protein concentrations of the cell lysates were determined and lysates containing equal protein were used for immunoprecipitation. Cells lysates were immunoprecipitated with mAbs against against *α*6 (43B-9B), *β*1 (PD52) or isotype matched control. Samples were resolved in 7.5% SDS–PAGE gel under nonreducing conditions and transferred to nitrocellulose membranes. Membranes were then probed with (**A**) anti-*α*6 integrin; (**B**) anti-*β*1 integrin antibodies and the interaction of *α*6*β*1 integrin was evaluated with ECL.

Ovarian cancer cell lines OVHS 1, PEO.36, OVCA 433 and HEY have moderate to high expression of *α*6, *α*v and *β*1 integrin (Table 1). In contrast, OVHS 1, PEO.36 and HEY cell lines lacked *β*4 integrin expression, while low expression of *β*4 integrin was observed in OVCA 433 cell line (Table 1). The expression of *α*v, *β*1 and *β*4 integrin (in OVCA 433 cell line only) remained unchanged in response to ascites after 48 h, while the expression of *α*6 integrin was enhanced in OVHS 1, PEO.36, OVCA 433 and HEY cell lines (Table 1). In contrast, no change in the expression of *α*6 integrin was observed in HOSE cells (Figure 1, Table 1). In order to determine if *α*6 integrin interacts with *β*1 integrin subunit and *vise versa*, we immunoprecipitated *α*6 and *β*1 integrin from HEY and OVCA 433 cell lines (Figure 2A, data shown for HEY). The immunoprecipates were immunoblotted with anti-*α*6 and anti-*β*1 integrin antibodies, and protein bands with relative identical mobility coimmunoprecipitating with *α*6 and *β*1 integrin were identified (Figure 2A and B). Immunoblots of *α*6 integrin immunoprecipitates also showed a band at 70–80 kDa, a protein previously identified as *α*6p, an incomplete form of *α*6 integrin missing the extracellular domain associated with ligand binding (Demetriou *et al*, 2004). *α*6p is not paired with *β*1 integrin subunit (Figure 2A and B) as has been previously shown for other cancer cells (Demetriou and Cress, 2004).

The expression of uPAR was enhanced in invasive OVCA 433 and HEY cell lines, while no such enhancement was observed in noninvasive and moderately invasive OVHS 1 and PEO.36 ovarian cancer cell lines and HOSE cells (Figure 1 and Table 1). Enhancement of uPAR correlated with an increase in the cell surface expression of uPA in OVCA 433 and HEY cells lines (Table 1) while no change in uPA expression was observed in HOSE, OVHS 1 and PEO.36 cell lines (Table 1). The change in the expression of *α*6 integrin and uPA/uPAR was investigated in OVHS 1, PEO.36, OVCA 433 and HEY cell lines with five different samples of ascites obtained from patients diagnosed with Stage 2 and Stage 3 ovarian cancer. These results indicate that soluble factors in ascites can affect the expression of *α*6 integrin and uPA/uPAR in ovarian cancer cells but not in HOSE cells.

### Immunohistochemical localisation of *α*6 and *β*1 integrin and uPAR in ovarian carcinomas

Immunohistochemical expression of *α*6 and *β*1 integrin and uPAR was evaluated in normal ovaries, benign, grade 1, 2 and 3 tumours. Of the eight benign tumours, six were of serous and two of mucinous origin. All eight grade 1 tumours were of endometriod subtype, while three of the grade 2 tumours were of serous origin and the remaining two were of endometriod subtype. Of the 13 grade 3 tumours, 10 were of serous, one endometrioid and two clear cell carcinoma subtype.

**Figure 3:**
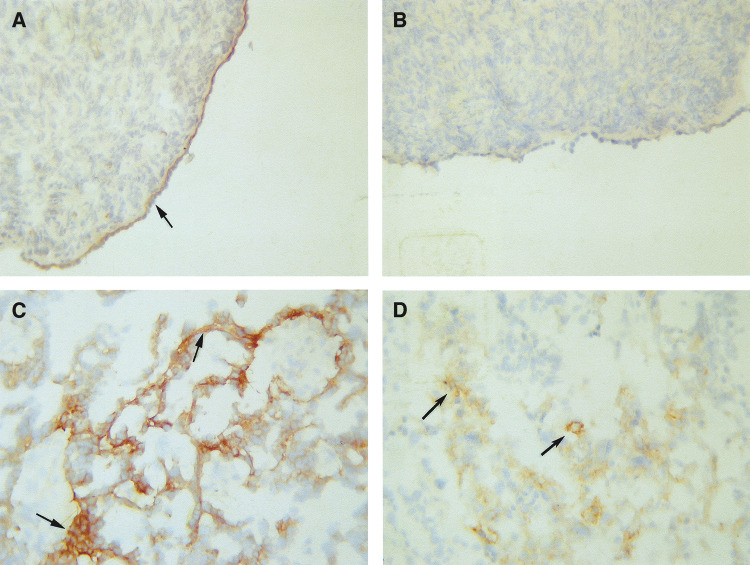
Expression of *α*6 integrin and uPAR in normal ovaries and ovarian tumours. Cryostat sections of ovarian tissues were stained by the immunoperoxidase method for the expression of *α*6 integrin and uPAR as discussed in the Materials and Methods section. (**A**) Normal ovary, arrow showing continuous basal expression of *α*6 integrin in the epithelium; (**B**) normal ovary, showing no expression of uPAR; (**C**) grade 3 serous ovarian tumour, arrows indicating irregular expression of *α*6 integrin in the basal epithelial and (**D**) grade 3 serous ovarian tumour, arrows indicating cytoplasmic epithelial staining of uPAR.

In the normal and benign ovarian tumours, the expression of *α*6 integrin was confined to the basal layer of epithelial cells which displayed continuous labelling (Figure 3A). When blood vessels were present on the section, staining of endothelial cells was also observed. In a few cases, staining of the medullary stroma of the normal ovaries was also evident. *α*6 integrin expression occurred in a scattered heterogenous fashion in ovarian tumours (Figure 3C). In all malignant tumours, the basal membrane reactivity was present in a discontinuous fashion. Blood vessel staining of endothelial cells was also evident in some tumour sections. *α*6 integrin epithelial staining was significantly higher in high-grade tumours compared to benign and grade 1 tumours and normal ovaries (*χ*^2^=27.41, df=12, *P*<0.05) (Table 2a). In contrast, the basement membrane *α*6 integrin expression was lower in high-grade tumours (*χ*^2^=16.85, df=12, *P*<0.05) while no significant differences in the blood vessel expression of *α*6 integrin was evident.

All normal and tumour specimens examined demonstrated extensive staining of *β*1 integrin subunit (data not shown). The expression of integrin was present in both epithelium and stroma with little or no difference in distribution between the different types or grades of tumour.

No expression of uPAR was evident in six normal ovaries examined (Figure 3B and Table 2b). The expression of uPAR was also absent in benign tumours. All ovarian malignant tumours showed some degree of epithelial staining for uPAR (Table 2b). Greater extent of staining was evident in advanced grade 3 tumours compared to grade 1/2 tumours (*P*<0.01) (Figure 3D). However, no staining of basement membrane and stroma was evident using this antibody.

### Proliferation of ovarian cancer cells in response to ascites

**Figure 4:**
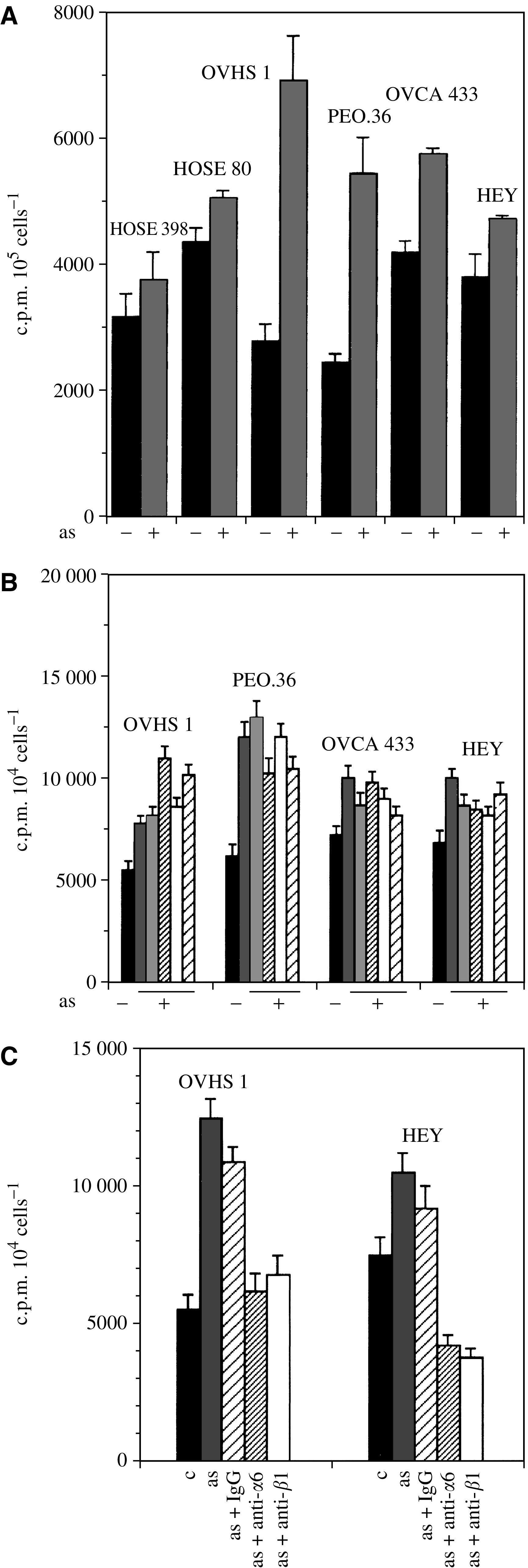
Effect of ascites on the proliferation of HOSE and ovarian cancer cells. The proliferative response of HOSE and ovarian cancer cells was determined on monolayer cultures and by 3D collagen gel system as described in Materials and Methods. (**A**) HOSE and ovarian cancer cell lines were grown as monolayers on 24-well plates and treated with ±ascites (3 mg ml^−1^) and the uptake of [^3^H]thymidine was measured over 16 h as described in Materials and Methods. The experiment was performed three times in triplicate. (**B**) Ovarian cancer cell lines grown in 3D collagen gel system were treated with five different ascites samples and their proliferation response was measured. The experiment was performed three times in triplicate. (**C**) Effect of *α*6 and *β*1 integrin inhibitory antibodies on the ascites-induced proliferation of OVHS1 and HEY cell lines in 3D collagen gel system.

As ascites is a rich source of soluble growth factors, we determined the proliferative response of HOSE and ovarian cancer cell lines in response to ascites in monolayer cultures over 24 h. The noninvasive OVHS1 and moderately invasive PEO.36 cell lines showed 2–2.5-fold increase in proliferation compared to 1.4–1.5-fold increase in invasive HEY and OVCA 433 cell lines (Figure 4A, *P*<0.01). No significant increase in proliferation was observed in HOSE cells (Figure 4A). To determine if ascites-induced enhancement in the proliferative response in monolayer cultures correlated with that in 3D collagen gels, the assay was repeated with five different ascites samples in 3D collagen gel system over 5 days. The noninvasive OVHS 1 and moderately invasive PEO.36 cell lines showed 1.5–2.2-fold (*P*<0.02) increase in the proliferative response compared to only 1.1–1.3-fold (*P*<0.02) increase in invasive OVCA 433 and HEY cell lines (Figure 4B). Inhibitory antibodies against *α*6 and *β*1 integrin (5 *μ*g ml^−1^) inhibited both ascites-induced and basal proliferation of noninvasive OVHS 1 and invasive HEY cancer cell lines (Figure 4C, *P*<0.01). No effect on proliferation was observed with control IgG. These results indicate that *α*6 and *β*1 integrin are involved in ascites-induced and normal proliferation of ovarian cancer cells.

### Adhesion of ovarian cancer cells in response to ascites

**Figure 5:**
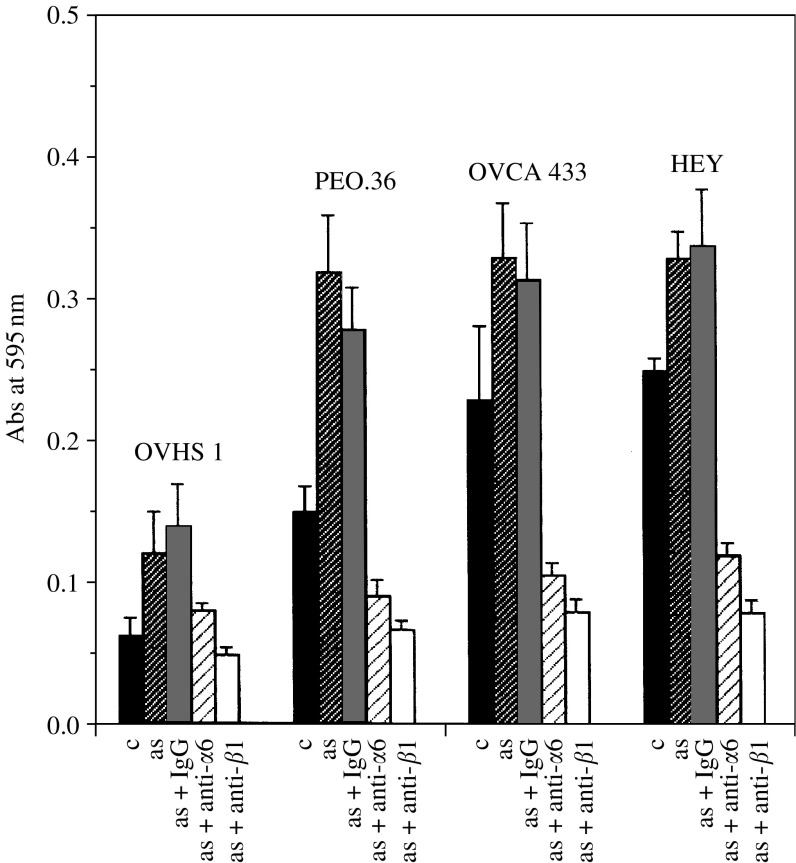
Effect of ascites on the adhesion of HOSE and ovarian cancer cell lines. The adhesive response of ovarian cancer cells in response to ascites was determined as described in Materials and Methods. Ovarian cancer cell lines were treated for 24 h with ±ascites (3 mg ml^−1^), cells were washed and adhesion of the cells was measured using 96-well plates. The experiment was repeated three times in triplicate.

To determine if the soluble growth factors in ascites have any effect on the adhesion of ovarian cancer cells, we investigated the adhesive response of HOSE and ovarian cancer cell lines in response to ascites. The four ovarian cancer cell lines used in the study showed enhanced adhesion when stimulated by ascites for 24 h (Figure 5). OVHS 1 and PEO.36 cells showed two-fold more adhesion compared to 1.4–1.5-fold adhesion (*P*<0.01) observed in OVCA 433 and HEY cell lines. Ascites-induced and basal adhesive response of the ovarian cancer cell lines was suppressed by inhibitory antibodies against *α*6 and *β*1 integrin (5 *μ*g ml^−1^) but not by control IgG (Figure 5, *P*<0.01). Ascites had no effect on the adhesion of HOSE cells under similar conditions (data not shown).

### Invasion of ovarian cancer cells in response to ascites

**Figure 6:**
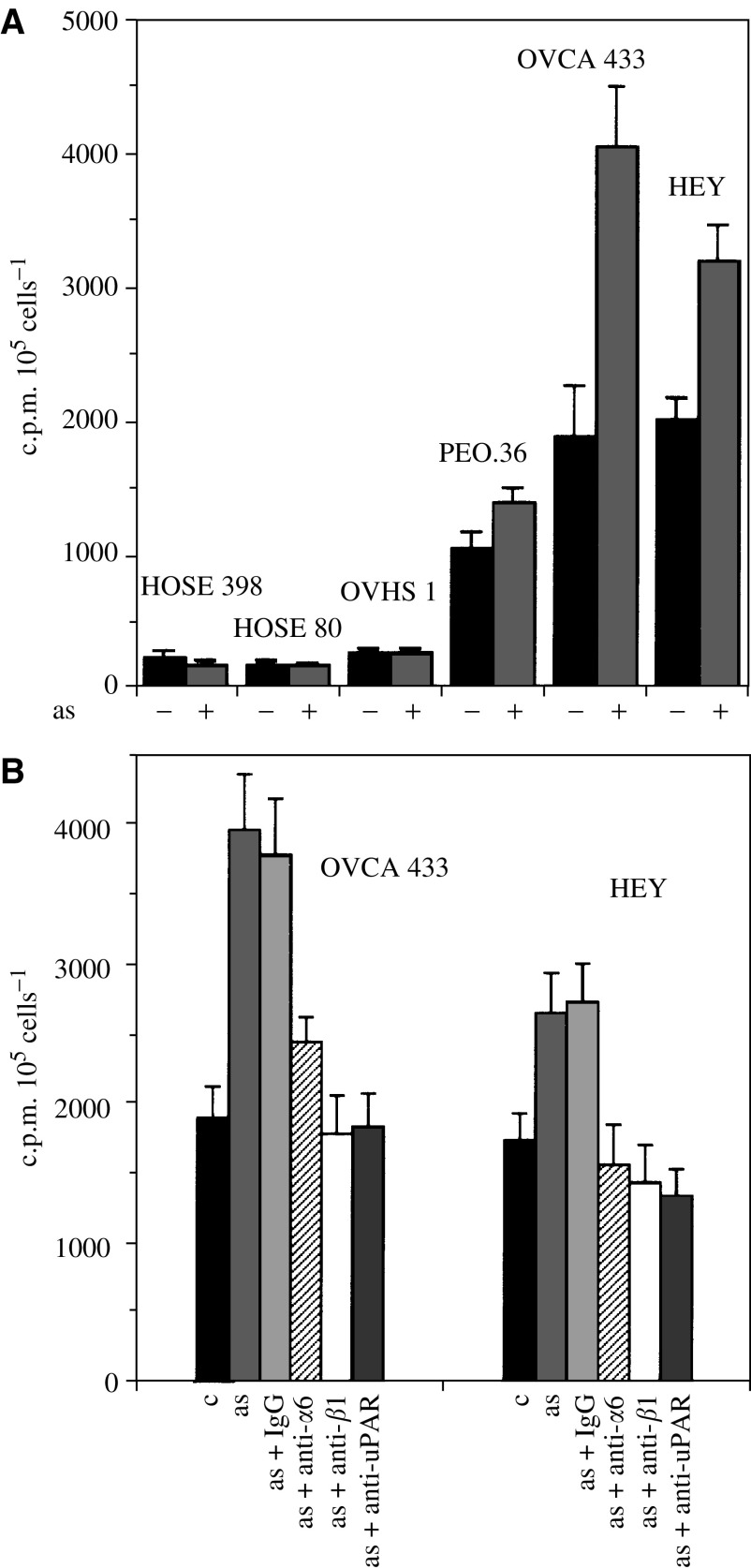
Effect of ascites on the invasion of HOSE and ovarian cancer cell lines. The invasive potential of HOSE and ovarian cancer cells was determined by Matrigel Boyden chamber as described in Materials and Methods. (**A**) HOSE and ovarian cancer cell lines were treated with ±ascites (3 mg ml^−1^) and their invasion capacity was measured in serum-free medium using 5% FBS as chemoattractant. The experiment was performed three times in triplicate. (**B**) Effect of *α*6, *β*1 integrin and uPAR inhibitory antibodies on the ascites-induced invasion of OVCA 433 and HEY cell lines.

Growth factors like epidermal growth factor (EGF), lysophosphatidic acid (LPA) and hepatocyte growth factor (HGF) are important constituents of ascites (Richardson *et al*, 2002) (Sandmann *et al*, 2003). As these growth factors have been shown to enhance the invasion of cancer cells (Ueoka *et al*, 2003), we compared the invasive potential of the four ovarian cancer cell lines stimulated for 24 h with ascites using Matrigel Boyden chambers. HOSE and OVHS1 cell lines showed no invasion capacity through the chambers irrespective of the presence or absence of ascites (Figure 6A). Even though PEO.36 cells were moderately invasive, invasive capacity was not significantly enhanced in response to ascites (Figure 6A). In contrast, OVCA 433 and HEY cell lines showed 1.5–2-fold enhanced invasion (*P*<0.01) in the presence of ascites. The invasiveness of OVCA 433 and HEY cell lines correlated with an increase in cell surface bound uPA and uPAR (Table 1). Ascites-induced invasiveness of OVCA 433 and HEY cell lines was inhibited by inhibitory antibodies against *α*6 and *β*1 integrin and uPAR (5 *μ*g ml^−1^) (Figure 6B, *P*<0.01) but there was no change in basal invasive response. Control IgG had no effect on the invasiveness of OVCA 433 and HEY cell lines. These data suggest that ascites-induced enhancement of *α*6 and *β*1 integrin and uPAR may have a role in enhancing the invasiveness of ovarian cancer cells.

### Ascites activates Ras and downstream Erk pathway

**Figure 7:**
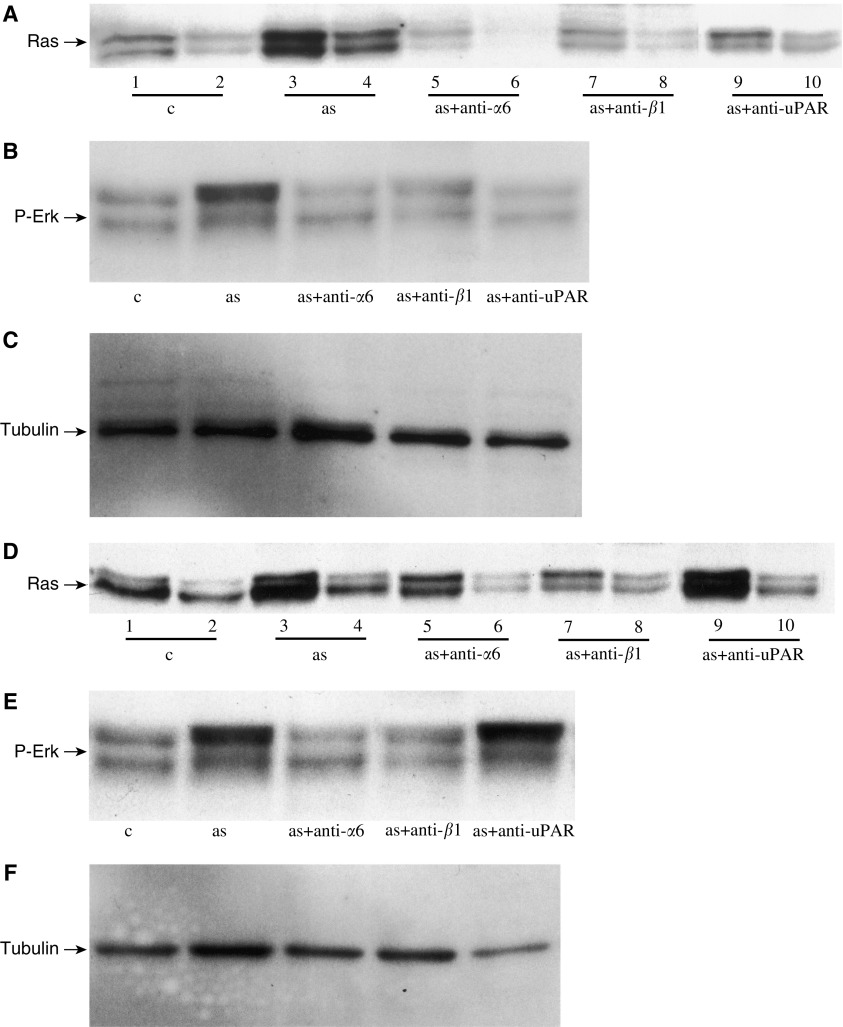
Effect of ascites on the activation of Ras and Erk pathway in HEY and OVHS 1 cell lines. Activation of Ras and downstream Erk was performed as described in Materials and Methods. (**A**, **D**) HEY and OVHS 1 cells were serum starved for 16 h and then treated with ascites in the presence or absence of inhibitory antibodies for 30 min. Cells were lysed and Ras-GTP and Ras-GDP were immunoprecipitated as described in Materials and Methods. Equal amount of protein was loaded in each lane. Lanes 1, 3, 5, 7 and 9 demonstrate expression of Ras-GTP, while lanes 2, 4, 6, 8 and 10 demonstrate the expression of Ras-GDP. (**B**, **E**) Effect of ascites and *α*6, *β*1 integrin and uPAR inhibitory antibodies on the ascites-induced activation of Erk in HEY and OVHS 1 cells. (**C**, **F**) *β*-Tubulin staining of the cell lysate to show protein loading.

Activation of Ras and downstream Erk pathway is involved in maintaining growth, adhesion and invasiveness of cancer cells (Ueoka *et al*, 2003). In order to assess if ascites can activate Ras/Erk pathway to regulate proliferative, adhesive and invasive functions of ovarian cancer cells, we investigated the effect of ascites on this pathway. Ascites enhanced the activation of Ras, as evidenced by the increased Ras-GTP levels in all four ovarian cancer cell lines studied (data shown for Hey and OVHS1 cell lines) (Figure 7A and D). Activation of Ras correlated with the activation of downstream Erk, indicating that ascites may induce activation of Erk through a Ras-dependent pathway (Figure 7B). In order to assess if *α*6*β*1 integrin and uPAR affect ascites-induced activation of Ras and downstream Erk, cells were treated for 30 min with the inhibitory antibodies (5 *μ*g ml^−1^) before being exposed to ascites for 30 min. Inhibition of *α*6 and *β*1 integrin and uPAR signalling pathway blocked activation of Ras and downstream Erk in HEY cell line (Figure 7A and B). Similar effects of anti-*α*6 and anti-*β*1 integrin were observed in OVHS 1 cell line (Figure 7D and E) but no effect of anti-uPAR antibody was observed on ascites-induced Ras and Erk activation in OVHS 1 cell line (Figure 7D and E). These data suggest that *α*6*β*1 integrin and uPAR expression have a defined role in sustaining ascites-induced activation of signalling pathways.

## DISCUSSION

The microenvironment created directly or indirectly by the tumours has an effect on the functional outcome of cancer cells within a tumour (Fidler, 2002). Ascites is a physiological manifestation of ovarian carcinoma arising from the tumour and also from the noncancer-bearing peritoneal surface of the ovary (Hirabayashi and Graham, 1970). It has long been known that factors that increase vascular permeability are present in ascites and contribute to its development (Zebrowski *et al*, 1999). These include angiogenic factors such as vascular endothelial growth factor (VEGF), HGF, basic fibroblast growth factor (bFGF), inteleukin-12, etc. and growth factors such as EGF, LPA, transforming growth factors *α* and *β* (TGF *α* and *β*), etc. (Zeimet *et al*, 1998). These soluble factors provide a unique microenvironment for ovarian cancer cells either encapsulated in the tumour or floating as a single-cell suspension or aggregates of cells in the peritoneal cavity or attached to the mesothelial lining of the peritoneum. Previous studies have concentrated in investigating the functional changes of ovarian cancer cells in response to individual growth factors present in ascites such as VEGF (Yabushita *et al*, 2003) or LPA (Bian *et al*, 2004). Yet, nothing is known about the effect of combination of all these ascites-associated soluble factors on the cellular growth and function of different population of ovarian cancer cells. In this study, we have focused in identifying some of the molecules that are regulated by ascites with a view to gain better understanding of the ascites-induced molecular mechanisms that may contribute to the progression and dissemination of ovarian carcinomas.

We demonstrate that the ovarian cancer cell lines undergo selective increase in *α*6 integrin subunit expression in ovarian cancer cells but not in HOSE cells. Ascites had no effect on the expression of *α*v and *β*1 integrins. No expression of *β*4 integrin was observed in three of the four ovarian cancer cell lines studied, while very low expression of *β*4 integrin was observed in OVCA 433 cells. *β*4 integrin expression in OVCA 433 cells remained unchanged in response to ascites. *α*6 integrin but not the *α*6p variant interacts with *β*1 integrin subunit in ovarian cancer cells. The lack of expression of *β*4 integrin in ovarian cancer cells is consistent with previous studies reporting deficiency of *β*4 integrin expression in malignant cells derived from the ascites of the ovarian cancer patients (Skubitz, 2002). As most ovarian cancer cell lines are derived from the ascites of cancer patients, the lack of *β*4 integrin subunit in these cell lines is not unexpected. These observations are consistent with those reported for prostate cancer as the progression of the cancer from intraepithelial neoplasia to invasive prostate carcinoma results in loss of *β*4 integrin expression and is replaced by alternative *α*6*β*1 integrin functions (Demetriou and Cress, 2004). Increase in *α*6 integrin expression has been reported previously for cells undergoing malignant transformation such as fibroblasts (Lin *et al*, 1993), mouse epidermal keratinocytes (Van Waes *et al*, 1995), hepatocytes (Begum *et al*, 1995), Lewis lung carcinoma cells and osteocarcinomas (Gomez *et al*, 1992). As ascites is one of the contributing factors in the dissemination of ovarian carcinoma, it would not be unreasonable to expect enhanced expression of *α*6 integrin in ovarian cancer cells in response to ascites.

Strong expression of *α*6 integrin was observed in the intact basement membrane underlying the surface epithelial layer of normal ovaries. In ovarian tumours, there was a loss of the regular basement membrane resulting in irregular staining of *α*6 integrin. In ovarian carcinomas, staining for *α*6 integrin was confined to irregular basal surfaces of the epithelial cells, where they were in contact with the basement membrane. Epithelial staining of *α*6 integrin was significantly higher in high-grade tumours compared to normal ovaries and benign tumours, while the staining of basement membrane was reversed. These results are consistent with expression of *α*6 integrin shown to correlate with the expression of basement membrane laminin in serous ovarian carcinoma (Skubitz *et al*, 1996). In another study, the expression of *α*6 integrin was shown to correlate with the degree of ovarian carcinoma tumour differentiation (Bottini *et al*, 1993). Our results, however, did not show such a correlation. As *α*6 integrin (combines with *β*1 or *β*4 integrin) is a major laminin receptor for adhesion in laminin-rich basement membranes, one can speculate that tumour-induced irregular patterns of basement membrane distribution can result in the loss of regular adhesion of cancer cells with subsequent exfoliation of cells into the peritoneum. As exfoliated ovarian cancer cells in the peritoneum require cell–cell or cell–ECM adhesion to grow and metastasise at sites within the peritoneum, the induction of microenvironment-induced expression of adhesion and metastasis-related molecules may serve to regulate the process of ovarian cancer growth, adhesion and invasion.

In this study, enhancement in the proliferation and adhesion of ovarian cancer cell lines in response to ascites was observed. However, these responses were significantly greater in noninvasive and moderately invasive ovarian cancer cell lines compared to invasive cell lines. Ascites, however, did not induce significant increase in the proliferation or adhesion of HOSE cells. Ascites also enhanced the invasiveness of invasive ovarian cancer cell lines and this enhancement in invasiveness correlated with the increment of uPA/uPAR expression. However, no enhancement in the invasiveness or increment in uPAR expression was observed in HOSE cells. These data indicate that enhancement of *α*6 integrin and uPA/uPAR in ovarian cancer cells occurs specifically in the presence of ascites without affecting normal ovarian epithelial cell function. Proinvasive properties of ovarian cancer ascites-derived membrane vesicles have been reported recently (Graves *et al*, 2004).

Recently, we have shown that ascites enhances the expression of integrin-linked kinase and its downstream Akt pathway in ovarian cancer cells but not in HOSE cells (Ahmed *et al*, 2003b). Moreover, lysophosphatidic acid has been shown to stimulate ovarian cancer cell migration via the Ras-MEK Kinase pathway (Bian *et al*, 2004). In this study, we demonstrate that ascites-induced proliferation, adhesion and invasion of ovarian cancer cells correlated with activation of Ras and downstream Erk pathway. Inhibition of *α*6 or *β*1 integrin or uPAR signalling pathway inhibited ascites-induced activation of Ras and Erk pathway with subsequent inhibition in proliferation, adhesion and invasion of ovarian cancer cells. These observations suggest that *α*6*β*1 integrin and uPAR play a significant role in ascites-regulated functions of ovarian cancer cells.

Integrins directly associate with uPAR to mediate cellular function (Wei *et al*, 1999). uPAR has been reported to associate with many signalling molecules and to mediate signal transduction (Aguirre Ghiso *et al*, 1999). Previously, we have shown loss of uPA/uPAR-mediated Erk activation with downregulation of uPAR expression in colon cancer cells (Ahmed *et al*, 2003a). uPA/uPAR interaction with *β*1 integrin has been shown to activate Erk pathway (Ahmed *et al*, 2003a) and disruption of this interaction can result in loss of adhesion and tumour progression in nude mice (van der Pluijm *et al*, 2001). The uPAR–integrin interaction is crucial as many integrin receptors activate intracellular signal pathways to fully activate cell survival and proliferation pathways (Miyamoto *et al*, 1996). Recently, we have shown that the cytoplasmic domain of *α*v*β*6 integrin interacts with Erk and that interaction is essential to maintain tumour growth in mice (Ahmed *et al*, 2002a). *β*1 integrin function is regulated by uPAR and downregulation of uPAR expression results in loss of uPAR/*β*1 integrin complex with subsequent inhibition of migration, matrix degradation and invasion of colon cancer cells (Ahmed *et al*, 2003a). *α*6 integrin/uPAR interaction has been shown in breast and prostate cancer cell lines (Demetriou and Cress, 2004), suggesting that signalling through *α*6 integrin and uPAR may be essential for ensuring cancer phenotype expression.

Primary tumours consist of subsets of cancer cells with different dominant phenotypic characteristic (Mortarini and Anichini, 1993). In ovarian cancer, this scenario is more complicated as the cancer is a compilation of different population of cells derived from different clonal origins either attached to a substratum (ovary or mesothelial layer) or floating in the peritoneum. In ovarian cancer patients, normal ovarian cells are also exposed to ascites. The fact that ascites has no effect on the proliferation and invasiveness of HOSE cells suggest that normal epithelial ovarian cellular function is not affected by ascites but rather ascites regulates the dominant characteristic of the subset of ovarian cancer cells. This scenario may provide functional advantage to ovarian cancer cells contributing to the progression of cancer. Hence, one can speculate that the presence of ascites will create a microenvironment to help subsets of genetically programmed ovarian cancer cells to perform their relevant functions through enhanced expression of adhesive and metastasis-related proteins. A more detailed understanding of the relative contribution of ascites-regulated molecules on subsets of ovarian cancer cells will promote better understanding of the biology of ovarian cancer and will also result in improved treatment. The concept of ascites being a cause rather then a manifestation of ovarian cancer progression warrants further consideration.

## Figures and Tables

**Table 1:** Expression of *α*6, *α*v, *β*1 and *β*4 integrins and uPA and uPAR in ovarian cancer and HOSE cells in response to ascites

**Antigen expression (MIF)**	**OVHS 1**	**PEO.36**	**OVCA 433**	**HEY**	**HOSE 80**
*α*6 integrin					
−as	67±6	1028±112	204±3	346±50	88±10
+as	176±24	1343±52	405±50	542±78	90±12
					
*αv* *integrin*					
−as	90±8	67±8	53±6	120±8	
+as	88±10	72±8	48±2	90±4	ND
					
*β1 integrin*					
−as	135±11	281±10	2308±88	1298±20	
+as	171±4	345±14	2147±28	1516±80	ND
					
*β4 integrin*					
−as	5±0	10±0	20±0	5±0	
+as	5±0	10±0	18±0	5±0	ND
					
*uPA*					
−as	10±0	5±0	12±0	28±4	10±0
+as	12±0	4±0	26±2	100±12	12±0
					
*UPAR*					
−as	12±0	5±0	12±0	34±15	10±0
+as	15±0	6±0	24±1	70±28	8±0

MIF=mean intensity of fluorescence; as=ascites; ND=not determined. The values are ±s.e.m. of experiments repeated with three different ascites. uPA=urokinase plasminogen activator; uPAR=urokinase plasminogen activator receptor.

**Table 2a:** Expression of *α*6 integrin in 40 normal and ovarian tumour tissues

**Histology**	**No. of patients**	**Epithelium**	**Basement membrane**	**Blood vessels**
Normal	6	0 (6)	4 (1), 5 (4)	4 (4), 5 (1)
Benign	8	0 (7), 3 (1)	4 (6), 5 (2)	3 (3), 4 (2), 5 (3)
Grade 1	8	0 (5), 2 (3)	4 (4), 5 (4)	3 (3), 3 (3), 5 (2)
Grade 2	5	0 (2), 2 (1), 3 (2)	3 (1), 4 (2), 5 (2)	2 (3), 3 (2)
Grade 3	13	2 (6), 3 (7), 4 (1)	2 (3), 3 (3), 4 (3), 5 (5)	2 (6), 3 (5), 4 (3)

The extent of *α*6 integrin expression was scored in a blind fashion as 0 (<10%), 1 (11–25%), 2 (26–50%), 3 (51–75%), 4 (76–90%) and 5 (>90%). For each tissue, four sections were studied and the whole tissue was analysed. The values in parentheses indicate the number of tissues with the extent of staining indicated in the respective columns. For each tissue, four sections were studied and the entire section was analysed. Epithelial expression of *α*6 integrin was significantly higher in high-grade tumours compared to benign and grade 1 tumours and normal ovaries (*χ*^2^=27.41, df=12, *P*<0.05). In contrast, the basement membrane *α*6 integrin expression was lower in high-grade tumours (*χ*^2^=16.85, df=12, *P*<0.05), while no significant differences in the blood vessel expression of *α*6 integrin was evident.

**Table 2b:** Expression of uPAR in 40 normal and ovarian tumour tissues

**Histology**	**No. of patients**	**Basement membrane**	**Epithelium**	**Blood vessels**
Normal	6	0	0	0
Benign	8	0	0	0
Grade 1	8	0	1 (5), 2 (3)	0
Grade 2	5	0	1 (2), 2 (3)	0
Grade 3	13	0	2 (5), 3 (4), 4 (4)	0

The extent of uPAR staining was scored as described for *α*6 integrin staining. Student's *t*-test was used to test the association between the extent of staining of uPAR and the histological grade of tumour. Greater extent of staining was observed in grade 3 tumours compared to grade 1/2 tumours (*P*<0.01).
